# Ergosterol-Induced Sesquiterpenoid Synthesis in Tobacco Cells

**DOI:** 10.3390/molecules17021698

**Published:** 2012-02-09

**Authors:** Fidele Tugizimana, Paul A. Steenkamp, Lizelle A. Piater, Ian A. Dubery

**Affiliations:** 1 Department of Biochemistry, University of Johannesburg, Auckland Park, Johannesburg, 2006, South Africa; Email: fideletu@gmail.com (F.T.); psteenkamp@csir.co.za (P.A.S.); lpiater@uj.ac.za (L.A.P.); 2 Drug Discovery and Development, CSIR Biosciences, Pretoria, 0001, South Africa

**Keywords:** ergosterol, metabolomics, *Nicotiana tabacum*, phytoalexins, secondary metabolites, sesquiterpenoids, UPLC-HDMS

## Abstract

Plants have the ability to continuously respond to microbial signals in their environment. One of these stimuli is a steroid from fungal membranes, ergosterol, which does not occur in plants, but acts as a pathogen-associated molecular pattern molecule to trigger defence mechanisms. Here we investigated the effect of ergosterol on the secondary metabolites in tobacco (*Nicotiana tabacum*) cells by profiling the induced sesquiterpenoids. Suspensions of tobacco cells were treated with different concentrations (0–1,000 nM) of ergosterol and incubated for different time periods (0–24 h). Metabolites were extracted with a selective dispersive liquid-liquid micro-extraction method. Thin layer chromatography was used as a screening method for identification of sesquiterpenoids in tobacco extracts. Liquid chromatography coupled to mass spectrometry was used for quantitative and qualitative analyses. The results showed that ergosterol triggered differential changes in the metabolome of tobacco cells, leading to variation in the biosynthesis of secondary metabolites. Metabolomic analysis through principal component analysis-scores plots revealed clusters of sample replicates for ergosterol treatments of 0, 50, 150, 300 and 1,000 nM and time-dependent variation at 0, 6, 12, 18 and 24 h. Five bicyclic sesquiterpenoid phytoalexins, capsidiol, lubimin, rishitin, solavetivone and phytuberin, were identified as being ergosterol-induced, contributing to the altered metabolome.

## 1. Introduction

Metabolites can be viewed as the end products of gene expression and define the biochemical phenotype of a cell or tissue [1,2,3]. Qualitative and quantitative analyses of large numbers of cellular metabolites provide a broad view of the biochemical status of an organism [4,5,6]. Metabolites are widely diverse in their respective chemical and physical properties (molecular weight, polarity, solubility, volatility) and it is currently impossible to extract and analyse all metabolites (metabolome) in a cell/organism in a single analysis [1,7,8,9,10]. However, different metabolomic strategies and approaches have been developed for different analyses. These include metabolite target analysis (qualitative and quantitative analysis of specific metabolites related to a specific metabolic reaction); metabolite fingerprinting (sample classification by rapid, global analysis); metabonomics (evaluation of tissues and biological fluids for changes in endogenous metabolite levels); and metabolite profiling (identification and quantification of a selected number of pre-defined metabolites, generally related to a specific metabolic pathway) [1,5,11]. A range of analytical platforms used in metabolomics includes gas chromatography-mass spectrometry (GC-MS), capillary electrophoresis-mass spectrometry (CE-MS), liquid chromatography-mass spectrometry (LC-MS), nuclear magnetic resonance spectroscopy (NMR), direct infusion mass spectrometry (DIMS), and Fourier transform-infrared (FT-IR)- and Raman spectroscopies [12,13,14,15,16].

As sessile organisms, plants are continuously threatened by a wide range of pathogens and insect herbivores. To defend themselves plants have preformed antimicrobial metabolites (phytoanticipins) to prevent or attenuate invasion by potential attackers [17,18,19,20,21]. Furthermore, plants have evolved sophisticated abilities to recognise their attackers and to translate this perception into an effective immune response. The primary immune response recognises conserved features of pathogens such as flagellin, chitin, glycoproteins, lipopolysaccharides and ergosterol. These elicitors are referred to as microbial/pathogen-associated molecular patterns (M/PAMPs). MAMPs are recognised by pattern recognition receptors (PRRs), which in turn initiate diverse downstream signalling events that ultimately result in the activation of a defence response that is called MAMP-triggered immunity (MTI) [20,22,23,24,25,26]. The inducible chemical defence arsenal of plants includes the antimicrobial phytoalexins. The final outcome of the pathogen:plant interaction depends ultimately on the balance between the ability of the pathogen to suppress the plant’s immune responses and the capacity of the plant to recognise the pathogen and to activate effective defences [27,28].

Ergosterol is the principal sterol of fungal plasma membranes, with an essential role in membrane stabilization and signalling [29]. Ergosterol does not occur in plants and is recognised by a plant cell as ‘non-self’ [30,31]. Research has shown that ergosterol acts as a MAMP molecule in tobacco and tomato plants, resulting in a MTI response. This defence reaction is characterised by production of reactive oxygen species (ROS), changes in ion fluxes, activation of defence genes and production of defence-related secondary metabolites [29,30,32,33].

The effect of ergosterol on plant secondary metabolism has not been thoroughly investigated. In the present study a metabolomic approach was utilised to elucidate and analyse changes in secondary metabolism of tobacco (*Nicotiana tabacum*) cells following ergosterol treatment. The work focuses on sesquiterpenoid as a class of defence-related metabolites. The phytoalexins isolated from plants within the *Solanaceae* are mostly bicyclic sesquiterpenoids [34,35]. The latter are considered to form part of defence-related secondary metabolites involved in plant:microbe interactions [36].

## 2. Results and Discussion

A viability assay, based on the ability of viable cells to reduce 2,3,5-triphenyltetrazolium chloride (TTC) [37], was used to determine if possible cell death occurred due to ergosterol treatment. No loss of cell viability was observed over the concentration range of 0–1,000 nM (data not shown); indicating that the observed responses are due to the treatment alone and possible secondary responses due to cell death can be excluded.

### 2.1. Dynamic Changes Occur in the Metabolome of Ergosterol-Treated Tobacco Cells

In order to investigate the ergosterol-induced changes in the metabolome of tobacco cells, concentration- and time studies were conducted. These studies served also to establish the optimal conditions for treatment of the cells, which were found to be 300 nM ergosterol and an 18 h incubation period (data not shown).

**Figure 1:**
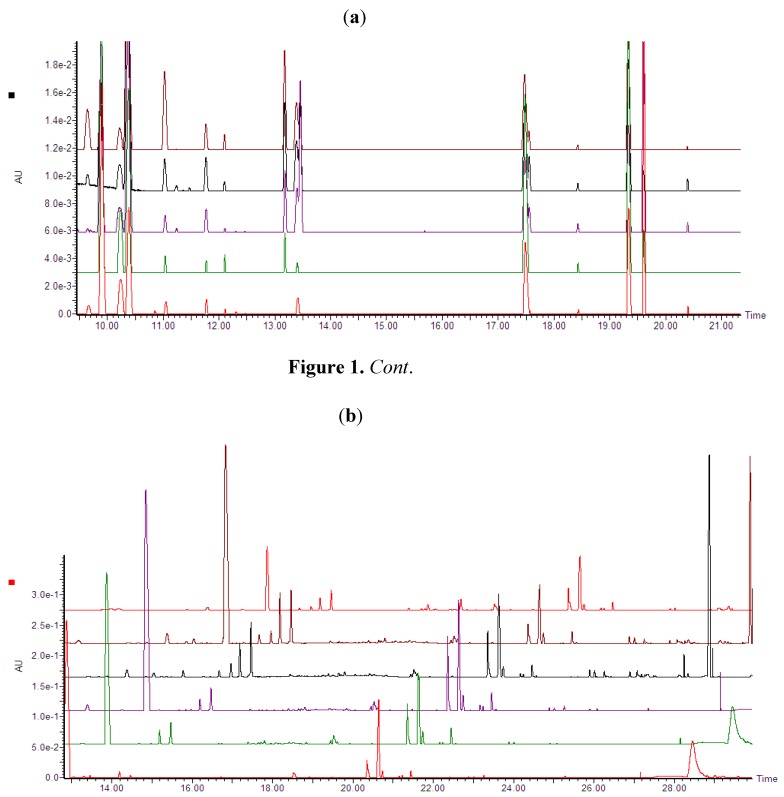
(**a**) UPLC-PDA chromatograms (photodiode array range: 200–500 nm) showing treatment-related variations (Concentration study). Dispersive liquid-liquid microextraction (DLLME) samples of tobacco cells treated with different ergosterol concentrations (from bottom to top: 0, 50, 150, 300 and 1000 nM) and incubated for 18 h. (**b**) UPLC-PDA chromatograms (photodiode array range: 200–500 nm) showing time-related variations. DLLME extracts of tobacco cells treated with 300 nM ergosterol and incubated for different time period (from bottom to top: 0, 6, 12, 18 and 24 h NT) and the top last chromatogram is a non-treated sample incubated for 24 h (24 h NT).

Ultra performance liquid chromatography coupled to high definition mass spectrometry (UPLC-HDMS) was used for the analyses. Inspection of the base peak intensities (BPI) chromatograms of both concentration- and time studies (Figure 1a,b) indicate clearly that ergosterol treatment induced differential metabolic changes as exemplified by increases or decreases in peak intensities, new peaks and peak suppression.

In addition to visual inspection of chromatograms, an unsupervised multivariate data analysis (MVDA), principal component analysis (PCA), was carried out to differentiate between ergosterol treatments. The MarkerLynx software was used to pre-process the UPLC-MS chromatographic data as described in the experimental section. The extracted data (quantified peaks), in a matrix format, were exported into SIMCA-P12 software for PCA modelling.

PCA reduces the dimensionality of the data without much loss of information and expresses the data in such a way as to identify and highlight the similarities and differences in systematic patterns and features of the data set [8,38,39,40,41]. PCA is based on the notion of latent variables; and using an orthogonal transformation procedure, the correlated variables are converted into uncorrelated variables called principal components (PCs). The PCA scores plot offers a visual image of sample variations from a global view, and the PCA loadings scatter plot permits the evaluation of the contribution that each ion mass makes to the total information of the analysed data. Since PCA is a non-parametric analysis, the generated model is independent of the user, hence unsupervised [42,43].

**Figure 2:**
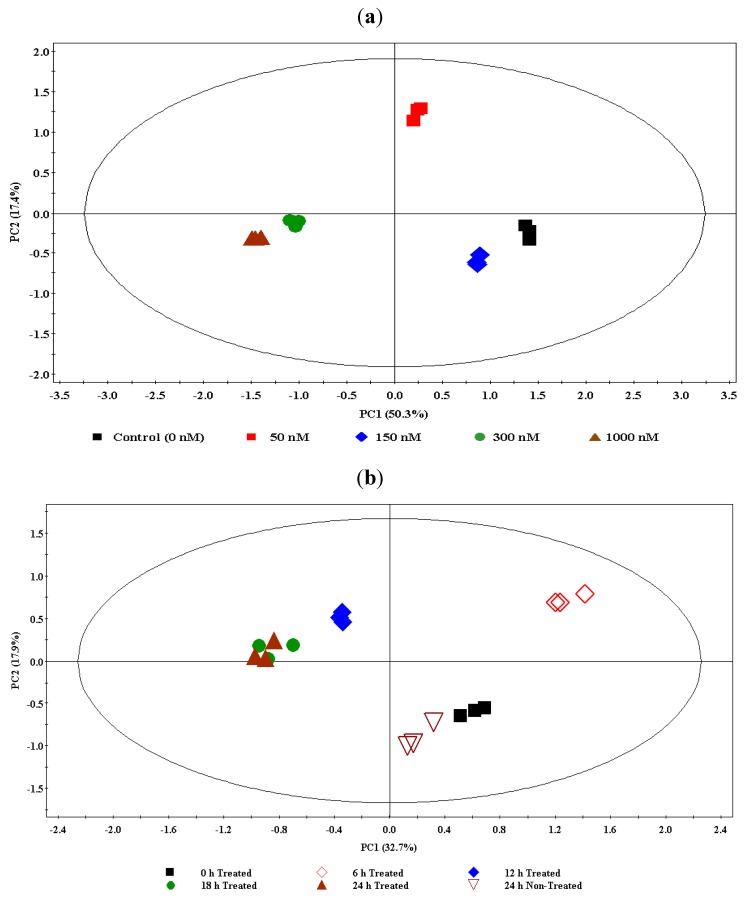
(**a**) The principal component analysis (PCA) of the UPLC-MS concentration study data (ESI positive): DLLME extracts of tobacco cells treated with 0 nM- (control), 50 nM-, 150 nM-, 300 nM-, and 1,000 nM- ergosterol and incubated for 18 h. The scores plot shows the clustering/separation of different treatments (0 nM/control, 50 nM, 150 nM, 300 nM, and 1,000 nM) with little variation within each group. (**b**) The PCA of the UPLC-MS time study data (ESI positive): DLLME extracts of tobacco cells treated with 300 nM ergosterol and incubated for different time period (0 h T–24 h T) and a non-treated sample incubated for 24 h (24 h NT).

For the concentration study data (ESI^+^), a nine-component model was computed and explained 92.4% of the variance. Using the first two principal components (PC1 and PC2, explaining 67.7% of the variance) for a scores plot, the samples were found to be differentially clustered into five groups corresponding to different ergosterol treatments (0–1,000 nM) with no significant intra-group variation (Figure 2a). The clusters corresponding to the 300 nM and 1,000 nM treatments are clustered close to each other, indicating that a near maximum response was reached at 300 nM. Thus, from this scores plot of PCA a clear ergosterol-induced metabolomic change is evidently depicted, indicating dosage-dependence and dynamic responses within the cells.

For time study data (ESI^+^), an eleven-component model was generated and explained 90.2% of the variance. The first two components (PC1 and PC2) explained 50.6% of the variance. A scores plot was constructed using PC1 and PC2, showing samples differentially clustered into different groups (Figure 2b): The extracts from non-treated samples (incubated for 24 h) are seen to group with the 0 h-incubated treated samples (treated with 300 nM ergosterol); the 6 h- and 12 h-incubated samples formed different and separate groups, but the 18 h- and 24 h-incubated samples are clustered together, indicating that the response is essentially complete at 18 h. The PCA models for ESI^−^-MS data of both concentration- and time studies are provided in the supplementary data (Figures S1-2).

The variation indicated by inspection of BPI chromatograms and explained by PCA, shows that tobacco cells respond to the perception of ergosterol and that this response is reflected by differential changes in the cellular metabolite profiles. These changes include variation in the levels of the constitutively expressed metabolites, and production of new metabolites. These ergosterol-induced metabolic changes can be described as part of the defence response of the tobacco cells following perception of ergosterol. No metabolomic study has been done before to investigate the effect of ergosterol on tobacco cell metabolism. The results of this metabolomic study, focused on the chloroform extractable metabolites, thus suggest that the metabolic variation observed in the tobacco cell suspensions in response to ergosterol, are linked to reprogramming of the metabolome. 

### 2.2. Ergosterol Induces Sesquiterpenoid Phytoalexins in Tobacco Cells

One of the difficulties that arise in metabolomic studies is the identification of the *de novo*-induced compounds due to both their very restricted amounts and the high complexity of the biological extracts [44]. Furthermore, in the case of plant:pathogen interaction studies, the induced metabolites have different rates of accumulation. Moreover, these metabolites may not be stable and can undergo bioconversion and degradation either by the plant or in the extraction process. Plants contain enzymes that degrade antimicrobial compounds, returning their levels to pre-infection or pre-stress concentrations after the infection or stress has been contained or accommodated [21,45]. Thus, the identification of induced metabolites remains a very challenging task.

In this study, mass spectrometry (in combination with UPLC) was used for identification of ergosterol-induced sesquiterpenoids. High performance thin layer chromatography (HPTLC) analysis, followed by vanillin/sulphuric acid detection, served as a positive control to screen for the presence of sesquiterpenoid phytoalexins in extracts as discussed below [46,47].

#### 2.2.1. HPTLC Fractionation of Extracts and Initial Characterisation

**Figure 3:**
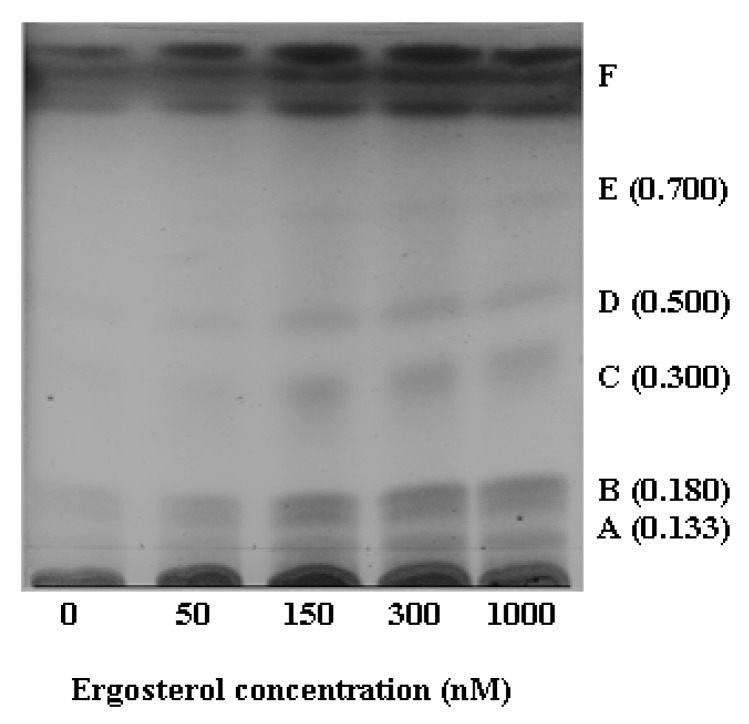
HPTLC image indicating differences between the DLLME chloroform extracts from control (non-treated) and treated cells. Chromatography was performed with chloroform: methanol (19:1, v/v) and detection with vanillin/sulphuric acid reagent.

HPTLC analysis of the chloroform extracts allowed partial characterisation of the multi-component cell extracts. The image of the HPTLC plate (Figure 3) shows the six spots obtained, with assigned letters A to F. The retardation factor (R_f_) value of each spot was determined. By comparison of these experimentally determined R_f_ values against the literature values [46], some of these spots were tentatively identified. Spot B (R_f_ = 0.18) was identified as capsidiol (the literature R_f_ = 0.18); spot C (R_f_ = 0.30) as rishitin (the literature R_f_ = 0.26); spot D (R_f_ = 0.50) as lubimin (the literature R_f_ = 0.48) and spot E (R_f_ = 0.70) as solavetivone (the literature R_f_ = 0.77) [46]. Spots A (R_f_ values of 0.13) and F (>one band) could not be identified based on their R_f_ values. Thus, as a screening method, HPTLC results provided an indicative separation of the constituents of the samples, showing the presence of the sesquiterpenoid phytoalexins in the ergosterol-treated samples. 

#### 2.2.2. UPLC-QTOF-ESI-MS based Metabolites Identification

**Figure 4:**
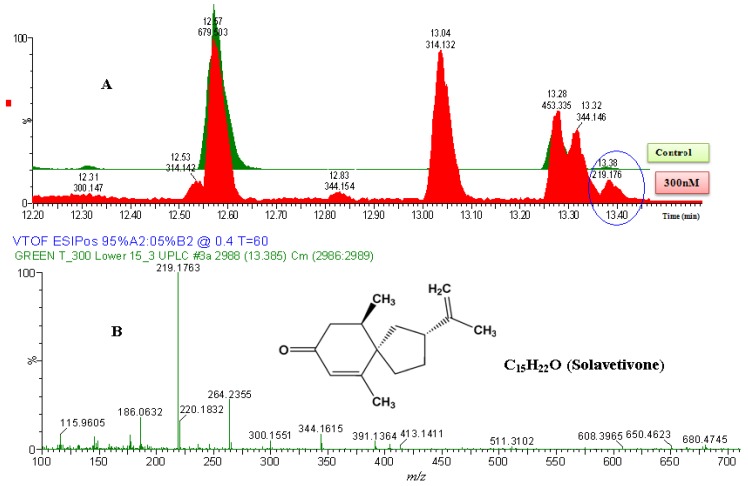
The identification of solavetivone. (**A**) The zoomed-in ESI^+^-MS TICs comparing non-treated sample (green) and 300 nM-treated sample (red). The encircled ion peak is present only in the treated sample. (**B**) MS-spectrum of the encircled peak, eluted at 13.38 min (219.1763 Da). Based on the spectrum of the extracted ion peak, the empirical formula calculated (and selected) was C_15_H_23_O, with i-FIT of 0.0 and DBE of 4.5. Since the ion was generated by ESI^+^ mode, the calculated empirical formula would contain one proton (H^+^) extra. The corrected empirical formula is thus C_15_H_22_O; and searching in databases (structural correlation to MS spectrum), the compound was putatively identified as solavetivone (C_15_H_22_O, 218.340 Da).

**Figure 5:**
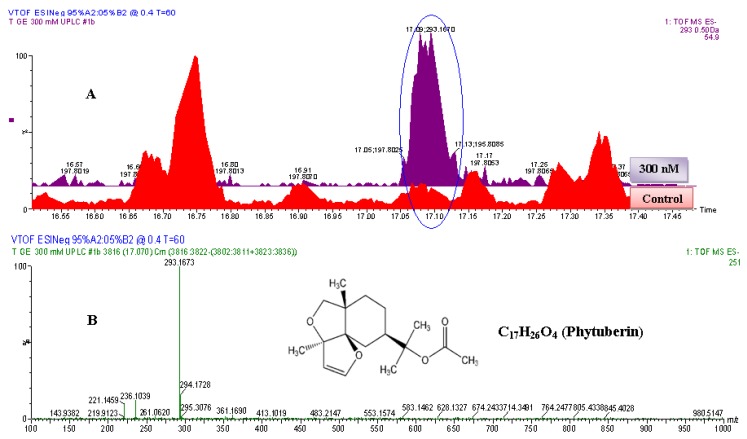
The identification of phytuberin. (**A**) The zoomed-in extracted ESI^−^-MS TICs comparing non-treated (control) and 300 nM-treated samples. The encircled ion peak is present only in the treated sample: the intensity of the peak is significantly higher in the 300 nM-treated sample than in non-treated (control) sample. (**B**) MS-spectrum of the extracted ion peak, eluted at 17.1 min (293.1673 Da). Based on the spectrum of the extracted ion peak, the empirical formula calculated (and selected) was C_17_H_25_O_4_, with i-FIT of 0.0 and DBE of 5.5. Since the ion was generated by ESI^−^ mode, the calculated empirical formula would contain one proton (H^+^) less. The corrected empirical formula is thus C_17_H_26_O_4_; and searching in databases (with structural correlation to MS spectrum) the compound was putatively identified as phytuberin (C_17_H_26_O_4_, 294.183 Da).

**Figure 6:**
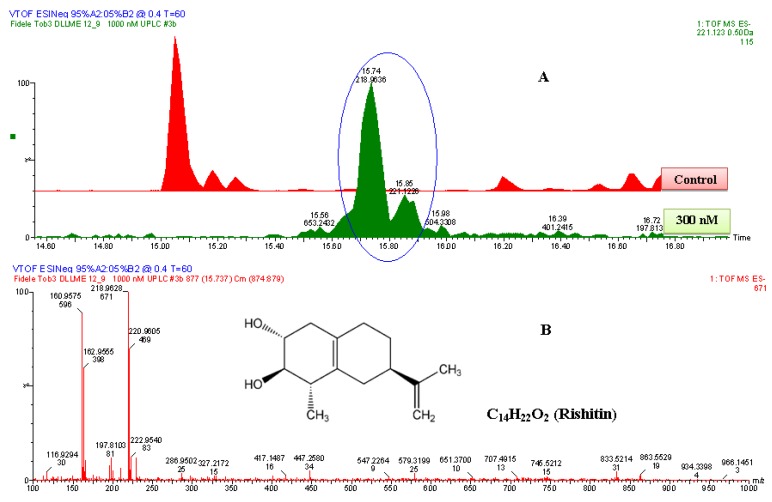
The identification of rishitin. (**A**) The zoomed-in extracted ESI^−^-MS TICs comparing non-treated (control) and 1000 nM-treated samples. The encircled ion peak is present only in the treated sample. (**B**) An ESI^−^-MS-spectrum of the ion peak of 221.122 Da that eluted at 15.85 min. Based on the spectrum of the ion, the empirical formula calculated (and selected) was C_14_H_21_O_2_, with i-FIT of 1.1 and DBE of 4.5. Since the ion was generated by ESI^−^ mode, the calculated empirical formula had one proton (H^+^) less. Thus, the corrected empirical formula is C_14_H_22_O_2_; and searching in databases (with structural correlation to MS spectrum), the compound was putatively identified as rishitin (C_14_H_22_O_2_, 222 Da).

The compound-identification approach used consisted of comparison of the BPI chromatograms (total ion chromatograms—TICs) of different treatment conditions, and extracting the ion peaks that show differences (either in intensities or presence/absence). The mass spectra of the extracted ion peaks were used to deduce the putative empirical formulae of the compounds. Databases such as Dictionary of Natural Products (www.dnp.chemnetbase.com) and ChemSpider (www.chemspider.com) were consulted for the compound identification. Three sesquiterpenoids: Solavetivone, phytuberin and rishitin were accordingly identified (Figure 4, Figure 5, Figure 6).

**Figure 7:**
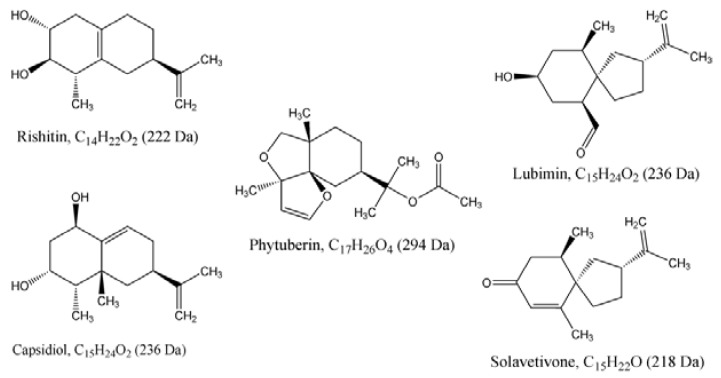
Chemical structures of the five sesquiterpenoid phytoalexins putatively identified in this study. All the sesquiterpenes, by definition, have the basic skeleton of C_15_ and are synthesised from a C_15_ molecule, farnesyl diphosphate (FPP), through the reactions catalysed by the enzymes prenyltransferase and sesquiterpene cyclases. The end products of these enzymes-catalysed reactions are various sesquiterpenoids with varying number of carbons such as C_15_, C_14_ and C_17_ [49,50,51,52].

Combining these MS-based identifications with the HPTLC analysis (Section 2.2.1.), the findings of this study evidently demonstrate that ergosterol induced the biosynthesis of five sesquiterpenoids (Figure 7 and Table 1) in tobacco cell suspensions. These putatively identified terpenoids are capsidiol, lubimin (HPTLC, Figure 3), phytuberin (UPLC-MS, Figure 4), rishitin and solavetivone (HPTLC/UPLC-MS, Figure 3, Figure 5 and Figure 6). As reported in previous studies [34,47,48], capsidiol, rishitin, lubimin and solavetivone are sesquiterpenoids found in plants within the *Solanaceae*, and are correlated with the defence response of plants to invading pathogens. 

#### 2.2.3. Ergosterol-Induced Metabolomic Reprogramming

The terpenoids are generally synthesized from isopentenyl diphosphate (IPP) and dimethylallyl diphosphate (DMAPP), which are the isomeric 5-carbon building block molecules. The IPP and DMAPP are the products of two independent pathways in plants, namely the mevalonate pathway operating in the cytosol and the glyceraldehyde-3-P/pyruvate (GAP/Pyr) pathway in plastids [50,51,52]. A series of enzyme-catalysed condensation reactions of IPP and DMAPP molecules leads to the biosynthesis of farnesyl diphosphate (FPP), which is a 15-carbon molecule from which sesquiterpenoids are synthesised in a sesquiterpene cyclase-catalysed reaction (Figure S3) [17,36,52,53,54].

The position of FPP in the terpenoid biosynthetic pathway is an important and potential regulatory branch point of sesquiterpenoid biosynthesis. Under normal conditions, the FPP is channelled toward the biosynthesis of sterol and prenyl-lipid moieties [53,55]. The elicitor-induced transient induction of 3-hydroxy-3-methylglutaryl coenzyme A (HMG CoA) reductase, involved in the synthesis of mevalonic acid and the *de novo* gene expression of sesquiterpene cyclase, a key enzyme in the biosynthesis of sesquiterpenoids have been reported [29]. Moreover, the enhanced expression of sesquiterpene cyclase was accompanied by the suppression of squalene synthase activity [55,56,57,58]. These responses all contribute to metabolic reprogramming and, potentially, an altered metabolome (see Supplementary Figure S3).

The presence of the five bicyclic sesquiterpenoids (phytuberin, solavetivone, capsidiol, lubimin and rishitin) in ergosterol-treated tobacco cells indicates that the changes to the metabolome are associated with a defensive function (‘defensome’) in response to elicitation by ergosterol as a M/PAMP molecule. These sesquiterpenoids have been previously reported to accumulate in plant cell suspension cultures or tissues challenged by pathogens, thereby providing an anti-microbial and fungitoxic environment [46,47,59,60,61]. These biotic stress-induced sesquiterpenoids have thus been called sesquiterpenoid phytoalexins [34,36,45,62], referring to anti-microbial compounds whose *de novo* synthesis and accumulation are induced in plants cells or tissues following plant : pathogen/elicitor interactions [49,62].

**Table 1:** Ergosterol-induced sesquiterpenoid phytoalexins in tobacco cells.

Name		Empirical formula	Molecular mass (Da)	Experimental mass (Da)	Extractionmethod	Identification method	i-Fit	ExperimentalR_f_ values	CAS number
**1**	Capsidiol	C_15_H_24_O_2_	236.178	---	DLLME	HPTLC	---	0.18	37208-05-2
**2**	Lubimin	C_15_H_24_O_2_	236.178	---	DLLME	HPTLC	---	0.50	35951-50-9
**3**	Phytuberin	C_17_H_26_O_4_	294.183	294.1673	DLLME	UPLC-MS	0.0	---	37209-50-0
**4**	Rishitin	C_14_H_22_O_2_	222.162	222.9768	DLLME	HPTLC/UPLC-MS	1.1	0.30	18178-54-6
**5**	Solavetivone	C_15_H_22_O	218.340	218.1763	DLLME	HPTLC/UPLC-MS	0.0	0.70	54878-25-0

## 3. Experimental

### 3.1. Reagents

All chemicals were of analytical grade quality and obtained from various international suppliers. The organic solvents used in this study were HPLC/UPLC grade chloroform (LabScan, Gliwice, Poland), methanol (LabScan) and acetone (Associated Chemical Enterprises, Johannesburg, South Africa). All equipment used was sterilised and cell treatment was carried out under sterile conditions.

### 3.2. Elicitation of Cells

A stock solution of 4.54 mM ergosterol, C_28_H_44_O (Sigma), was prepared in acetone, and *Nicotiana tabacum* cv Samsun cell suspensions were cultivated as previously described [63]. Three days after subculture, cells were treated by adding specific volumes of the stock solution of ergosterol to aliquots of cells suspensions with continuous shaking at 80 rpm at 25 °C. For concentration studies, ergosterol was added to final concentrations of 0–1,000 nM, for an 18 h incubation period, and non-treated cell suspensions were used as negative controls. For the time study, cell suspensions were treated with 300 nM ergosterol and incubated for 0–24 h, and a non-treated sample, incubated for 24 h was included as a control. Three biological replicates were used.

### 3.3. Cell Viability Assay

A viability assay, based on the ability of viable cells to reduce 2,3,5-triphenyltetrazolium chloride (TTC) [37], was used to determine if possible secondary responses due to treatment-induced cell death occurred. Briefly, cell suspensions were treated with different concentrations of ergosterol (0–1,000 nM) and incubated for 18 h. After incubation, the excess medium was removed by filtration, and the cells (0.4 g) were incubated in 0.6% TTC solution (17.9 mM in 0.05 M phosphate buffer, pH 7.5, 5 mL) for 3 h with shaking in the dark at 25 °C. Following incubation with TTC, the cells were pelleted by centrifugation at 5,100 rpm for 10 min, washed with dH_2_O (5 mL) and centrifuged again as above, discarding the supernatant. The red triphenylformazan was extracted from the pelleted cells by homogenisation in 96% ethanol (5 mL) for 30 s. The homogenate was centrifuged at 12,000 *× g* for 10 min to pellet the cell debris and the absorbance of the supernatant measured at 485 nm.

### 3.4. Extraction of Secondary Metabolites — Phytoalexins

The defence-related secondary metabolites from tobacco are generally sesquiterpenoids that exhibit aromatic and non-polar properties [17]. Thus the main extraction method developed to extract these metabolites was the dispersive liquid-liquid microextraction (DLLME) technique. DLLME is based on the equilibrium distribution process of the target analytes between sample solution and extraction solvent. It involves a ternary component system: water (dH_2_O)/disperser solvent/extraction solvent, where the extraction solvent and disperser solvent are rapidly injected into the aqueous sample solvent. The mixture is then mixed and a cloudy solution (water/disperser solvent/extraction solvent) is formed in the test tube. After centrifugation, the denser extraction solvent is collected in the bottom of the conical tube [64].

In this study, methanol and chloroform were used as disperser and extraction solvents respectively. DLLME was carried out as follows: After induction and incubation, the medium was filtered using a Buchner funnel and the cells collected. Two grams of cells were re-suspended in 100% methanol (20 mL) to quench enzyme activity and homogenised using an UltraTurrax homogenizer. The homogenates were centrifuged at 5,100 rpm for 7 min at 25 °C. The supernatants were placed into clean 50 mL round-bottom flasks, and the methanol evaporated to 1 mL at 50 °C using a Buchi Rotavapor R-200. To this, crude aqueous extract (1 mL), chloroform (200 µL) and analytical grade methanol (100 µL) were added. The mixture was vortexed for 30 s and centrifuged at 15,000 *× g* for 6 min at 25 °C in a microcentrifuge tube. Using a syringe, the extraction layer (bottom chloroform layer) was removed and filtered through 0.22 µm filters (Millipore), and placed in glass HPLC vials. The chloroform extracts were kept at −20 °C until further analysis. 

### 3.5. HPTLC Fractionation of Extracts and Initial Characterisation

HPTLC analysis of the chloroform extracts allowed partial fractionation and identification of the extracted metabolites. Ten µL of the chloroform extracts were applied to silica gel G60 F254 glass plates (Merck) and the HPTLC plates developed with chloroform-methanol (19:1 v/v). Since most of these non-polar secondary metabolites are non-chromogenic compounds, the plates were sprayed with a vanillin-sulphuric acid reagent in order to locate and visualise the separated sesquiterpenoids on the chromatogram. It was prepared by mixing methanol (25 mL), vanillin (0.7 g), and concentrated sulphuric acid (250 µL). The sprayed HPTLC plate was heated in an oven at 130 °C and evaluated regularly until spots could be visualised.

### 3.6. Ultra Performance Liquid Chromatography-High Definition Mass Spectrometry (UPLC-HDMS) Analyses

UPLC-HDMS analysis was done on a Waters Acquity UPLC coupled in tandem to a Waters photodiode array (PDA) detector and a SYNAPT G1 HDMS mass spectrometer (Waters, Manchester, UK). Chromatographic separation of the extracts was done utilising a Waters CSH C18 column (150 mm × 2.1 mm, 1.7 µm) thermostatted at 60 °C. A binary solvent mixture was used consisting of water (eluent A) containing 10 mM formic acid (natural pH of 2.3) and acetonitrile (Romil Chemistry, UK) (eluent B). The initial conditions were 95% A at a flow rate of 0.4 mL min^−1^ and kept constant for 2 min. A gradient was introduced to change the chromatographic conditions to 5% A at 22 min. The conditions were kept constant for 3 min to flush the column where after the column was returned to initial conditions at 27 min and allowed to equilibrate for 3 min. The run time was 30 min and the injection volume was 5 µL. The PDA detector was scanned between 200 and 500 nm (1.2 nm resolution) and set for collecting 20 spectra s^−1^. Each sample was injected and analysed three times (three technical replicates), to account for any analytical variability.

The SYNAPT G1 mass spectrometer was used in V-optics and operated in electrospray mode to detect the compounds of interest. Leucine enkephalin (50 pg mL^−1^) was used as reference calibrant to obtain typical mass accuracies between 1 and 3 mDa. The mass spectrometer was operated in both positive and negative mode with a capillary voltage of 2.5 kV, the sampling cone at 17 V and the extraction cone at 4 V. The scan time was 0.1 s covering the 100 to 1,000 Da range. The source temperature was 120 °C and the desolvation temperature was set at 450 °C. Nitrogen gas was used as the nebulisation gas at a flow rate of 800 L h^−1^. The software used to control the hyphenated system and perform all data manipulations was MassLynx 4.1 (SCN 704).

### 3.7. Data Analysis

In comparison to traditional univariate statistical methods, the MVDA models are well suited to provide ways of handling confounding and covariance patterns (both within and between variables), which are found in complex and multi-dimensional data sets from metabolomic studies [39,65]. An unsupervised MVDA, PCA modelling, was performed. ESI positive and negative raw data (from UPLC-ESI-TOFMS) were extracted using MassLynx XS software and analysed with MarkerLynx^TM^ software (Waters Corporation, Mildford USA). The MarkerLynx software extracts the raw LC-MS data and produces a matrix of Rt-*m/z* variable pairs, with the *m/z* peak intensity for each sample. MarkerLynx software parameters were set to analyse the 2–26 min retention time range of the chromatogram, mass range 100–700 Da, mass tolerance 0.01 Da, mass window 0.05 Da and a retention time window of 0.20 min. The data matrix obtained from MarkerLynx processing was also exported to the SIMCA-P12 software for PCA modelling. The data were Pareto-scaled. PCA scores plots were used to explain variations in the samples [40]. 

## 4. Conclusions

To our knowledge, no metabolomic profiling study has been previously conducted to investigate the effects of ergosterol on plant secondary metabolism. Previous work regarding the isolation and characterisation of sesquiterpenoid phytoalexins used an approach targeted at specific compounds with inducers other than ergosterol. Results obtained in this study indicate that perception of the fungal sterol, ergosterol, acting as a ‘non-self’ MAMP molecule, induces significant and dynamic metabolomic alterations in tobacco cell suspensions. These changes include the activation of the terpenoid pathway leading to *de novo* biosynthesis of five sesquiterpenoid phytoalexins putatively identified as capsidiol, lubimin, phytuberin, rishitin and solavetivone.

## Supplementary Materials

Supplementary materials can be accessed at: http://www.mdpi.com/1420-3049/17/2/1698/s1.
