# Supplementary material for: Ergosterol-Induced Sesquiterpenoid Synthesis in Tobacco Cells
Source: Molecules. 2012 Feb 9;17(2):1698–715. doi: 10.3390/molecules17021698 (PMC6268458; doi:10.3390/molecules17021698)

**Supplementary Materials**

**Figure S1.** The principal component analysis (PCA) of the UPLC-MS concentration study data (ESI negative): DLLME extracts of the tobacco cells treated with 0 nM-(control), 50 nM-, 150 nM-, 300 nM-, and 1,000 nM- ergosterol and incubated for 18 h. The PCA score plot shows the clustering/separation of different treatments (0/control, 50 nM, 150 nM, 300 nM, and 1,000 nM); almost no significant variation within each group. 50 nM and 150 nM are clustered close to each other, same as for 300 nM and 1,000 nM. The control is significantly different from the rest.


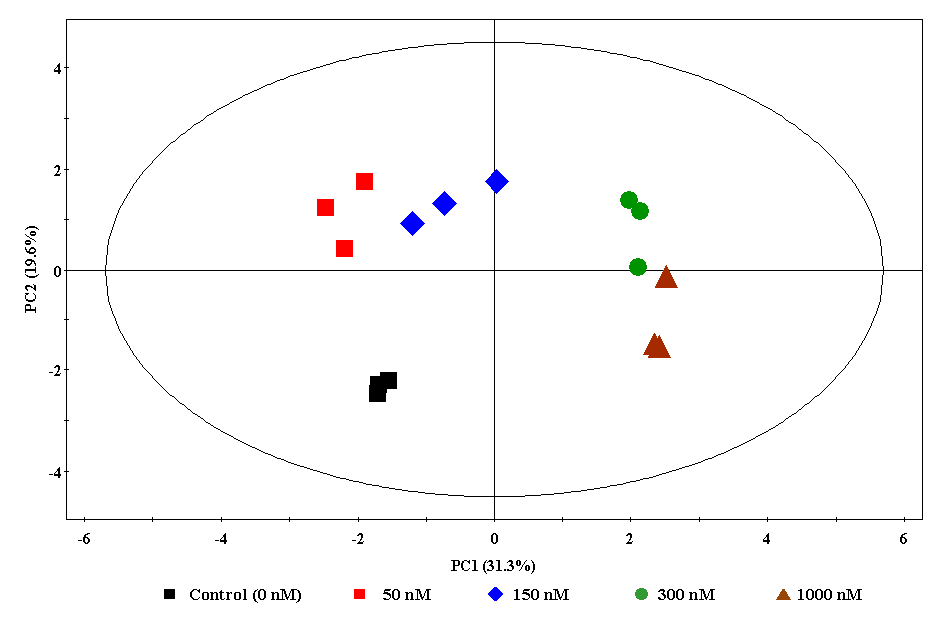


**Figure S2.** The principal component analysis (PCA) of the UPLC-MS time study data (ESI negative): DLLME extracts of the tobacco cells treated with 300 nM ergosterol and incubated for different time period (0 h T–24 h T) and a non-treated sample incubated for 24 h (24 h NT). The 0 h treated and 24 h non-treated samples clustered together (black and dark red colours); and the 18 h and 24 h treated samples grouped together (green and brown colours).


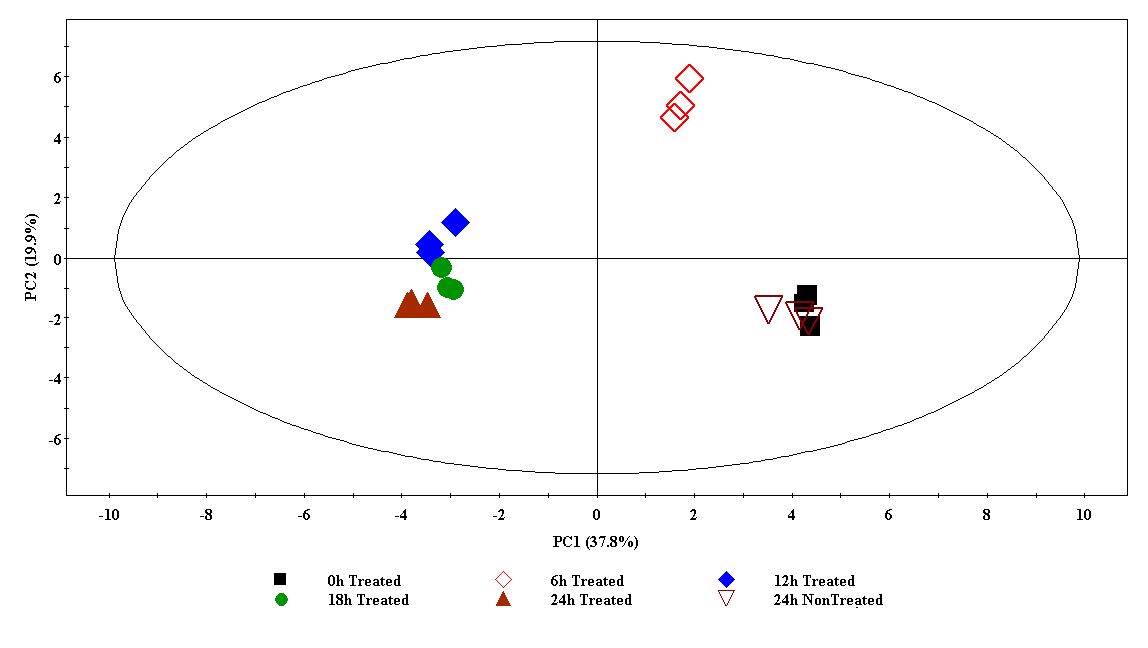


**Figure S3:** A simplified schematic representation of the biosynthesis of terpenoids and their cellular localisation. Two independent pathways, mevalonate pathway (in cytosol) and glyceraldehyde-3-P/pyruvate pathway (in plastids) generate the 5-carbon building block molecules: isopentenyl diphosphate (IPP) and dimethylallyl diphosphate (DMAPP). Farnesyl diphosphate (FPP) is formed from the enzyme-catalysed condensation reactions of IPP and DMAPP. FPP, a 15-carbon molecule is the building block of sesquiterpenoids.


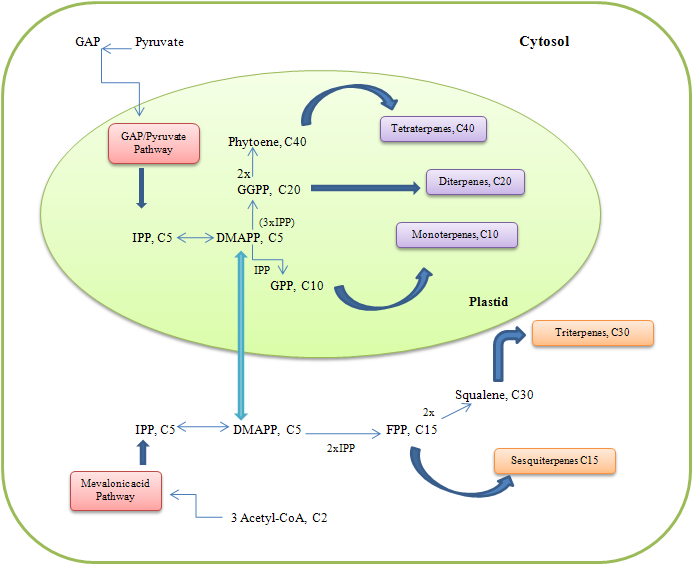

Supplement: Supplementary file 1 [file molecules-17-01698-s001.docx]
